# Fibrinogen-Like Protein 1 Serves as an Anti-Inflammatory Agent for Collagen-Induced Arthritis Therapy in Mice

**DOI:** 10.3389/fimmu.2021.767868

**Published:** 2021-12-16

**Authors:** Wen-Wei Lin, Kai-Wen Ho, Hsiang-Han Su, Tien-Fang Fang, Shey-Cherng Tzou, I-Ju Chen, Yun-Chi Lu, Mu-Shen Chang, Yun-Chen Tsai, En-Shuo Liu, Yu-Cheng Su, Yen-Tseng Wang, Tian-Lu Cheng, Hsin-Kai Huang

**Affiliations:** ^1^ Department of Laboratory Medicine, School of Post Baccalaureate Medicine, College of Medicine, Kaohsiung Medical University, Kaohsiung, Taiwan; ^2^ Department of Laboratory Medicine, School of Medicine, College of Medicine, Kaohsiung Medical University, Kaohsiung, Taiwan; ^3^ Graduate Institute of Medicine, College of Medicine, Kaohsiung Medical University, Kaohsiung, Taiwan; ^4^ Department of Medical Research, Kaohsiung Medical University Hospital, Kaohsiung, Taiwan; ^5^ Drug Development and Value Creation Research Center, Kaohsiung Medical University, Kaohsiung, Taiwan; ^6^ Department of Biotechnology, Kaohsiung Medical University, Kaohsiung, Taiwan; ^7^ Department of Biological Science and Technology, National Yang Ming Chiao Tung University, Hsinchu, Taiwan; ^8^ Department of Biomedical Science and Environmental Biology, Kaohsiung Medical University, Kaohsiung, Taiwan; ^9^ Department of Biochemistry, School of Post Baccalaureate Medicine, College of Medicine, Kaohsiung Medical University, Kaohsiung, Taiwan; ^10^ Department of Medical Laboratory, Kaohsiung Armed Forces General Hospital, Kaohsiung, Taiwan

**Keywords:** fibrinogen-like protein 1 (FGL1), lymphocyte-activation gene-3 (LAG-3), rheumatoid arthritis (RA), T cell, collagen-induced arthritis (CIA) model

## Abstract

Fibrinogen-like protein 1 (FGL1) was recently identified as a major ligand of lymphocyte-activation gene-3 (LAG-3) on activated T cells and serves as an immune suppressive molecule for regulation of immune homeostasis. However, whether FGL1 has therapeutic potential for use in the T cell-induced the autoimmune disease, rheumatoid arthritis (RA), is still unknown. Here, we attempted to evaluate the effect of FGL1 protein on arthritis progression. We also evaluated potential adverse events in a collagen-induced arthritis (CIA) mouse model. We first confirmed that soluble Fgl1 protein could specifically bind to surface Lag-3 receptor on 3T3-Lag-3 cells and further inhibit interleukin (IL-2) and interferon gamma (IFNγ) secretion from activated primary mouse T cells by 95% and 43%, respectively. Intraperitoneal administration of Fgl1 protein significantly decreased the inflammatory cytokine level (i.e., IL-1β and IL-6) in local paw tissue, and prevented joint inflammation, cellular infiltration, bone deformation and attenuated collagen-induced arthritis progression *in vivo*. We further demonstrated that exogenous Fgl1 does not cause obvious adverse events during treatment by monitoring body weight and liver weight, and assessing the morphology of several organs (i.e., heart, liver, spleen, lung and kidney) by pathological studies. We expect that Fgl1 protein may be suitable to serve as a potential therapeutic agent for treatment of RA or even other types of T cell-induced autoimmune or inflammatory diseases in the future.

## Introduction

Rheumatoid arthritis (RA) is an autoimmune disease caused by chronic inflammation in joint synovium, leading to bone erosion, joint deformation, limitation of mobility and decreased quality of life of patients (1, 2). It affects about 1% of population worldwide and is more prevalent in women, smokers, and those with a family history of the disease (1–3). Multiple immune cell types are found in inflammatory synovial joints of RA patients, such as macrophages, dendritic cells, T cells and B cells (2) and several studies have reported that T cells may play a central role in RA progression (2, 4–6). Thomas and Klarenbeek’s group indicated that CD4^+^ T cell are enriched and highly expanded in the synovial tissue of early RA disease (7, 8). The pro-inflammatory cytokine-interleukin 17 (IL-17) produced by CD4^+^ Th17 cells was shown to induce bone resorption in RA patients (9) and other Th17-produced cytokines (e.g., IL-6, IL-1β, IL-23, etc.) can also recruit neutrophils into synovial tissue to sustain chronic inflammation (10). Local CD4^+^ T cells have also been proposed to be a prominent driver of humoral immunity in RA patients and induced antibody against citrullinated protein and rheumatoid factor (RF), which are the two most prevalent autoantibodies in RA (11). These studies suggest that different subtype of T cells may contribute to RA progression in different ways. Thus, inhibition of T cell activity may be a potential therapeutic strategy for attenuating progression of RA.

Fibrinogen-like protein 1 (FGL1), also known as hepassocin, is a hepatocyte-derived secreted protein. It belongs to the fibrinogen family that can induce hepatocyte proliferation and recover liver injury by activating epidermal growth factor receptor (EGFR)/EGFR kinase (ERK) and the Src-dependent pathway (12–14). Recently, it was reported that FGL1 is highly expressed in several types of human cancer (e.g., lung cancer, prostate cancer, melanoma, gastric cancer, etc.) and is associated with resistance to programmed death-1 (PD-1)/programmed death ligand 1 (PD-L1)-blockage therapy and poor prognosis of cancer patients (15, 16). Wang and colleagues suggested that FGL1 is a major immune ligand for lymphocyte-activation gene 3 (LAG-3) (15), an inhibitory immune checkpoint protein on activated T cells, and is responsible for its T cell inhibitory function through an unclear mechanism (15). Wang et al. further demonstrated that FGL1 can inhibit the activity and growth effect of T cells and disrupt the interaction between FGL1 and LAG-3 through genetic ablation or monoclonal antibody blockage to significantly improve T cell responses and promote anti-tumor immunity (15). This implies that FGL1 may serve as a novel anti-inflammatory agent; however, its therapeutic potential for RA still unknown.

In this study, we attempted to evaluate the therapeutic potential of FGL1 protein on arthritis progression in a collagen-induced arthritis (CIA) mouse model. We first generated recombinant mouse Fgl1-Fc protein to assess the binding ability of Fgl1 with mouse Lag-3 receptor and investigated its inhibitory effect on primary T cell activity. We further evaluated the therapeutic efficacy of Fgl1 protein to disease progression in the CIA mouse model through monitoring the arthritis score, histological score and the expression of inflammatory cytokines in inflamed foot tissue. We also evaluated potential adverse events by assessing the body weight and the morphology of several organs (e.g., heart, lung, liver, etc.). This is first study to investigate the therapeutic potential of FGL1 for treatment of an auto-inflammatory disease and the findings presenting in this study may pave the way for an alternative therapeutic option for RA.

## Materials and Methods

### Cells, Animals and Reagents

Mouse fibroblast cell line NIH-3T3 was purchased from American Type Culture Collection (USA) and cultured in Dulbecco’s modified Eagle’s medium (DMEM; Sigma-Aldrich, St Louis, MO, USA) supplemented with 10% (v/v) bovine calf serum (BCS; Thermo Fisher Scientific, Waltham, MA, USA) and 100 units/mL penicillin and streptomycin (Invitrogen, Calsbad, CA) at 37°C in a humidified atmosphere of 5% (v/v) CO_2_. BALB/c mice were purchased from the National Laboratory Animal Center, Taipei, Taiwan. DBA/1J mice were kindly provided by Dr. Shey-Cherng Tzou (Department of Biological Science and Technology, National Yang Ming Chiao Tung University, Hsinchu, Taiwan). Animal experiments were carried out in accordance with institutional guidelines and approved by the Animal Care and Use Committee of Kaohsiung Medical University, Kaohsiung, Taiwan (IACUC: 106117). Mouse interleukin (IL)-2 ELISA kit and Carboxyfluorescein succinimidyl ester (CFSE) were purchased from Invitrogen (Calsbad, CA) and mouse interferon gamma (IFNγ) ELISA kit was purchased from R&D Systems (Minneapolis, MN, USA). Bovine serum albumin (BSA) and coomassie brilliant blue were purchased from Sigma-Aldrich (St. Louis, MO, USA). Mouse anti-his antibody was purchased from Millipore (Billerica, MA, USA). Rabbit anti-mouse Fgl1 antibody was purchased from OriGene Technologies (Rockville, MD, USA). Fluorescein (FITC)-conjugated goat anti-mouse IgG Fc and FITC-conjugated goat anti-rabbit IgG Fc antibodies were from Jackson Immunoresearch Laboratories (West Grove, PA, USA). Polybrene and puromycin were purchased from Sigma-Aldrich (St. Louis, MO, USA). Recombinant mouse Fgl1 protein by tagging the histidine tag was purchased from Sino Biological (Beijing, China). RIPA lysis buffer, phenylmethylsulfonyl fluoride (PMSF) and Protease Inhibitor Cocktail were purchased from Merck (Kenilworth, NJ, USA).

### Plasmid Construction and Expression

The complementary DNA (cDNA) coding for the mouse Fgl1 and Lag-3 genes were cloned through polymerase chain reaction (PCR). Primers used in the cloning of mouse Fgl1 and Lag-3 were as follows: Fgl1 sense, 5’- aaaaagcttgccaccatgggaaagatttacagcttcg-3’; Fgl1 antisense, 5’- tttgtcgacaataatatttggaataaaatcacttgg-3’; Lag-3 sense, 5’- aaagctagcgccaccatgagggaggacctgctc-3’; Lag-3 antisense, 5’- tttggcgcgcctcagagctgcctgggc-3’. The mouse Fgl1 sequence was subcloned into expression vector, pLNCX-mouse IgG1 Fc, by use of HindIII and SalI restriction sites. The mouse Lag-3 gene was subcloned into pLKO_AS3w lentiviral vector by use of NheI and AscI restriction sites and the pLKO_AS3w-mouse Lag-3 was further used to generate pseudotyped lentivirus. The pLNCX-Fgl1-mFc plasmid was transfected into and expressed in NIH-3T3 cells by LipoFectamine 2000 (Invitrogen, Calsbad, CA). Two days after transfection, the culture medium was collected and used to analyze the binding ability of Lag-3 receptor by flow cytometry.

### Generation of NIH-3T3-Lag-3 Cells by Lentiviral Transduction

Pseudotyped lentiviruses were generated by co-transfecting pLKO_AS3w-mouse Lag-3 with pCMVΔR8.91 or pMD.G (Academia Sinica, Taipei, Taiwan) in 293T cells by LipoFectamine 2000 (Invitrogen, Calsbad, CA). Two days after transfection, the culture medium was filtered, mixed with 8 μg mL^−1^ Polybrene (Sigma-Aldrich), and added to NIH-3T3 cells. Following lentiviral transduction, the cells were selected in 2 μg mL^−1^ puromycin-containing medium and sorted on a fluorescence-activated cell sorting (FACS) sorter (Beckman Coulter, Brea, CA) to generate NIH-3T3-Lag-3 cells that stably expressed mouse Lag-3 on their surface.

### Flow Cytometry

Surface expression of the mouse Lag-3 receptor was measured by staining the 3T3-Lag-3 cells with 1.25 μg mL^−1^ rat anti-mouse Lag-3 monoclonal antibody (eBioscience, Waltham, MA, USA) in phosphate-buffered saline (PBS) containing 0.05% (w/v) BSA on ice. After removal of unbound antibodies, cells were incubated with 3.75 μg mL^−1^ FITC-conjugated goat anti-rat IgG Fc (Jackson Immunoresearch Laboratories) in PBS containing 0.05% (w/v) BSA on ice. After extensive washing, the surface fluorescence of the viable cells was measured on a guava easyCyte flow cytometry system (Merck, Kenilworth, NJ, USA). The Fgl1 binding activity of membrane-tethered Lag-3 receptor was determined by incubating the NIH-3T3 or NIH-3T3-Lag-3 cells with supernatant of pLNCX-Fgl1-mFc plasmid-transfected NIH-3T3 cells. After removal of the supernatant, cells were incubated with 3.75 μg mL^−1^ FITC-conjugated goat anti-mouse IgG Fc (Jackson Immunoresearch Laboratories) in PBS containing 0.05% (w/v) BSA on ice. After removal of unbound antibodies by extensive washing in cold PBS containing 0.05% (w/v) BSA, the surface fluorescence of the viable cells was also measured on a guava easyCyte flow cytometry system.

### Isolation and Activation of Mouse Primary T Cells

Mouse T cells were isolated from splenocytes of wild-type female BALB/c mice by Pan T cell isolation kit, a LS Column and a MidiMACS Separator (Miltenyi Biotec, Bergisch Gladbach, North Rhine-Westphalia, Germany). The isolated T cells were then activated by using T cell Activation kit (Miltenyi Biotec, Bergisch Gladbach, North Rhine-Westphalia, Germany). In brief, 1×10^6^ T cells were incubated with anti-mouse CD3 and anti-mouse CD28 antibody-coated beads in the presence or absence of 5 μg/ml Fgl1 recombinant protein and cultured in 48-well plates at 37°C for 3 days. The culture supernatant was obtained at 72 h following stimulation, and the levels of IL-2 (Invitrogen, Calsbad, CA) or IFNγ cytokines (R&D systems, Minneapolis, MN) were measured using ELISA kits according to the manufacturer’s manual process.

### Animal Studies

Male 8 to 12-week-old DBA/1J mice were subcutaneously (s.c.) immunized with bovine type II collagen (Chondrex, Woodinville, WA, USA) emulsified in Freund’s complete adjuvant (Chondrex, Woodinville, WA, USA) in the tail on day 0, then intraperitoneally (i.p.) boosted with bovine type II collagen on day 21 (17). Arthritis scores were based on a previously described scoring system (18). The mice with a clinical score of greater than or equal to 1 were intraperitoneally treated with phosphate-buffered saline (PBS), 10 μg/kg or 50 μg/kg Fgl1 recombinant protein 3 times at 2 day intervals. Mice were sacrificed at the end of experiment and organs, including paws, joints, heart, liver, spleen, lung and kidney were collected and fixed in 4% paraformaldehyde for further tests. Tissues were embedded in paraffin for sectioning, and H&E staining and toluidine blue staining were performed to evaluate the phenotype or inflammatory response of tissue and cartilage. Bone tissue samples of the joint were decalcified with 0.5 M EDTA solution and embedded in paraffin to obtain slices with a thickness of 5 μm. The tissue embedding, sectioning, H&E staining and toluidine blue staining were conducted by Biosidsco Inc. (Kaohsiung, Taiwan). Cartilage damage scores were based on a previously described scoring system (19). Further, paw tissue was homogenized by using tissue grinding pestle and RIPA lysis buffer with protease inhibitor and centrifuged to collect the supernatant. The concentrations of IFNγ, IL-1β, IL-2, IL-6, IL-17A and TNFα in the paw tissues were measured using MILLIPLEX MAP magnetic bead panel assay (Merck, Kenilworth, NJ, USA) following the manufacturer’s instructions.

### Real Time Quantitative PCR

Total RNAs of paw tissue were extracted by TRIzol reagent (Invitrogen, Calsbad, CA) and reverse transcripted by Super-Script III reverse transcriptase (Invitrogen, Calsbad, CA). cDNA were amplified with iQ™ SYBR Green Supermix (Bio-Rad, Hercules, CA), and quantitative PCR was performed using a Real-time PCR 7500 (Applied Biosystems, Waltham, MA). Samples were normalized to glyceraldehyde-3-phosphate dehydrogenase (GAPDH) expression and showed in fold change (2^-ΔΔCt^). All of the primer sequences are provided in **Supplementary Table 1**.

### Statistical Analysis

Data are presented as mean ± SEM. All the readings were background adjusted by subtracting the absorbance of a blank control in the ELISA procedures. Results were analyzed *via* either the one-way or two-way ANOVA analysis of variance to compare the statistical significance of the differences between the controls and samples. Statistical analysis was performed using the GraphPad Prism v.6 and data were considered significant at a P value of less than 0.05.

## Results

### Binding Function of Fgl1 to Lag-3 Receptor

**Figure 1:**
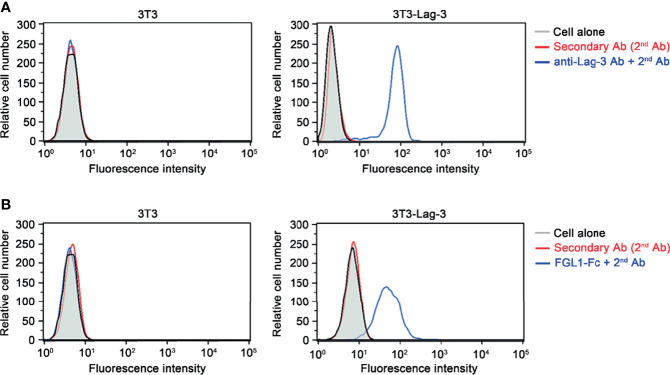
The binding of Fgl1 to Lag-3 receptor. NIH-3T3-Lag-3 cells (3T3-Lag-3 cells) that stably express full-length mouse Lag-3 on NIH-3T3 cells (3T3 cell) were generated. **(A)** The membrane expression of Lag-3 receptor on 3T3 (left panel) or 3T3-Lag-3 (right panel) were analyzed by flow cytometry using anti-mouse Lag-3 monoclonal antibody, followed by secondary antibody (blue), or secondary antibody alone (red). Gray, cells alone; Red, 2^nd^ Ab; Blue, anti-Lag-3 Ab and 2^nd^ Ab. **(B)** The binding ability of Fgl1-Fc was evaluated by staining both 3T3 (left panel) and 3T3-Lag-3 (right panel) with Fgl1-Fc expressing supernatant and secondary antibody, or secondary antibody alone (red). Gray, cells alone; Red, 2^nd^ Ab; Blue, FGL1-Fc and 2^nd^ Ab.

To analyze the binding function of Fgl1 protein to Lag-3 receptor, we constructed cDNA of mouse Fgl1 gene upstream of the Fc domain from mouse immunoglobulin G1, subcloned into pLNCX expression vector and expressed by NIH-3T3 cells (3T3 cells). The supernatant of pLNCX-Fgl1-Fc-transfected 3T3 cells was then incubated with 3T3-Lag-3 cells, which stably express mouse Lag-3 receptor on their cell membrane through lentiviral transduction, following staining with mouse IgG Fc specific secondary antibody and detection of the fluorescent signal by flow cytometry. **Figure 1** shows that mouse Lag-3 receptor stably expressed on the cell membrane of 3T3-Lag-3 cell but not 3T3 cells (**Figure 1A**) and Fgl1-Fc can specifically recognize Lag-3 as compared with the control group (**Figure 1B**), suggesting that Fgl1 recombinant protein can specifically interact with Lag-3 receptor.

### Inhibition Effect of Fgl1 on T Cell Activity

**Figure 2:**
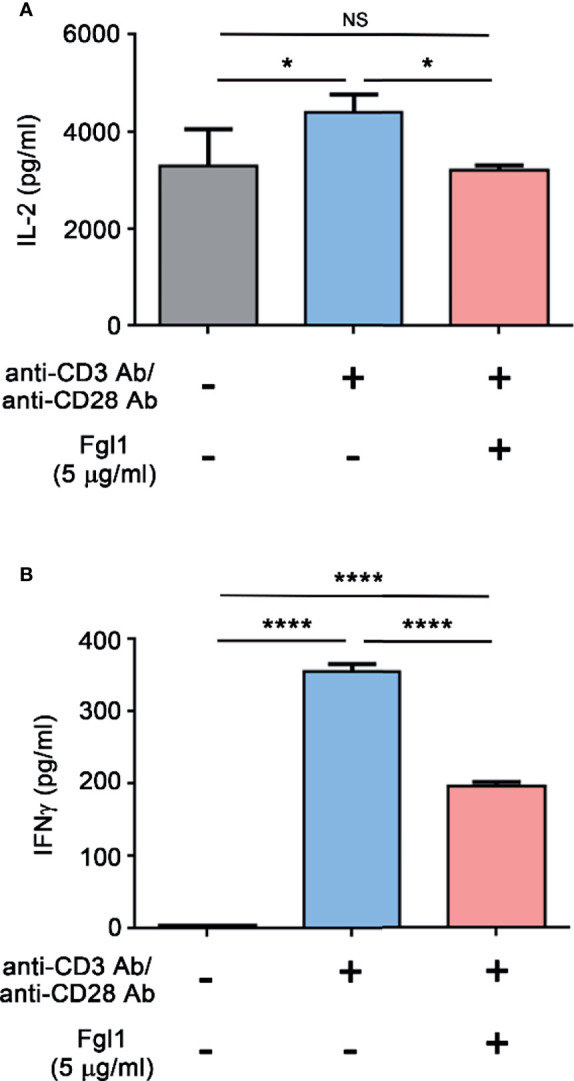
Fgl1 inhibition of T cell activity. Primary mouse T cells were incubated with anti-CD3/anti-CD28 antibody-coated beads and cultured in the presence or absence of Fgl1 recombinant protein (5 μg/ml) for 3 days. The **(A)** IL-2 or **(B)** IFNγ level in the supernatant was analyzed by antibody-based ELISA. All data are the mean ± SEM of triplicate independent experiments (n = 3). Statistical significance was calculated by One-way ANOVA. NS, not significant; *P < 0.05; ****P < 0.0001 when compared between each indicated group.

In order to obtain sufficient quantity and quality of Fgl1 protein, we used Fgl1 recombinant protein that purchased from Sino Biological. Inc. (**Supplementary Figure 1**) and investigated the inhibitory effect of Fgl1 on primary T cell activity. We treated activated primary mouse T cells with Fgl1 recombinant protein and monitored the expression level of cytokine markers of T cell activation (i.e., IL-2 and IFNγ). We first isolated mouse primary T cells from splenocytes of BALB/c mice through magnetic beads, then stimulated T cells with anti-CD3/anti-CD28 antibody-coated beads and cultured them in the presence or absence of 5 μg/ml Fgl1 recombinant protein. The cytokine markers (i.e., IL-2 and IFNγ) of T cell activation were analyzed by ELISA. As shown in **Figure 2**, Fgl1 recombinant protein could significantly suppress IL-2 production by over 95% (*P* < 0.05, **Figure 2A**) and IFNγ production by 43% (*P* < 0.001, **Figure 2B**) from activated primary T cells as compared with the untreated group. To further analyze the impact of Fgl1 on cell proliferation of activated T cells, we labeled primary T cells with carboxyfluorescein diacetate succinimidyl ester (CFSE) and stimulated T cells with anti-CD3/anti-CD28 antibody-coated beads cultured in the presence or absence of 5 μg/ml Fgl1 recombinant protein. The fluorescent signal was detected by flow cytometry. However, the CFSE dilution assay results showed that there was no significant difference in cell proliferation between the Fgl1-treated and untreated groups (**Supplementary Figure 2**). These results indicate that Fgl1 suppresses cell activity but not cell proliferation of T cells.

### Therapeutic Effect of Fgl1 on Arthritis Progression in a Collagen-Induced Arthritis (CIA) Mouse Model

**Figure 3:**
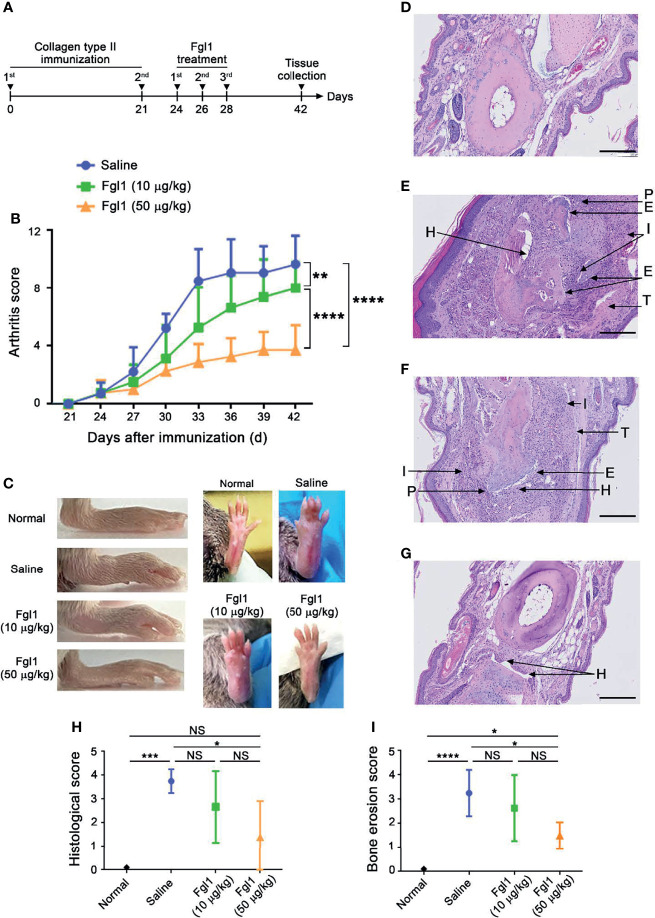
Therapeutic efficacy of Fgl1 protein in a collagen-induced arthritis (CIA) mouse model. **(A)** DBA/1J male mice were induced with arthritis by treating with type-II collagen at day 0 and 21. The collagen-induced arthritis (CIA) mouse model was intraperitoneally injected with normal saline (n=5), 10 (n=6) or 50 μg/kg (n=6) Fgl1 protein, respectively, at day 24, 26, and 28, and organs were collected at day 42. **(B)** Clinical score of the normal saline (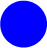), 10 (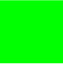) or 50 μg/kg (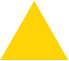) Fgl1 protein group was monitored every 2 days to evaluate the therapeutic efficacy. The values are mean ± SEM, and the statistical significance was calculated by Two-way ANOVA. The asterisks indicate a significant difference. **P < 0.01; ****P < 0.0001. **(C)** Macroscopic images of inflamed joints in each group are shown. Normal, normal DBA/1J mice; Saline, normal saline. Representative HE stained tissue sections of the hind paw in **(D)** normal DBA/1J mice, CIA mouse model after treatment with **(E)** normal saline, **(F)** 10 or **(G)** 50 μg/kg Fgl1 protein for 3 doses. The **(H)** histopathology score and **(I)** bone erosion score of the paw in saline- and Fgl1-treated CIA mouse model. The values are mean ± SEM, and the statistical significance was calculated by One-way ANOVA. The asterisks indicate a significant difference. *P < 0.05; ***P < 0.001; ****P < 0.0001. Representative histopathology images at 20× magnification. Bars = 200 μm. E, bone erosion; H, synovial hyperplasia; P, pannus; T, tendonitis; I, infiltration of inflammatory cells. NS, not significant.

**Figure 4:**
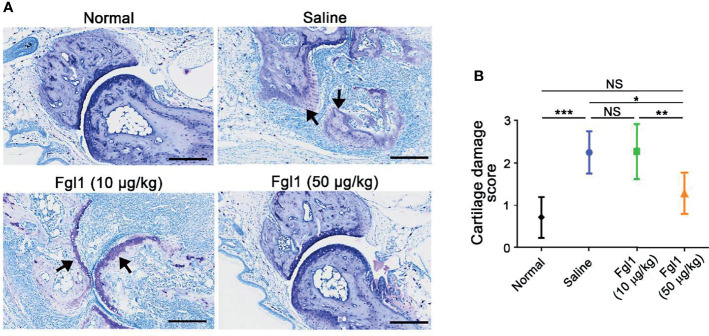
Toluidine blue staining to assess articular cartilage damage of Fgl1 protein in a collagen-induced arthritis (CIA) mouse model. (A) Representative toluidine blue stained tissue sections of the hind paw in normal DBA/1J mice (n=5), CIA mouse model after treatment with normal saline (n=5), 10 (n=6) or 50 μg/kg (n=6) Fgl1 protein for 3 doses. The **(B)** cartilage damage score of the paw in normal DBA/1J mice (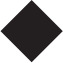), normal saline- (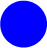), 10 (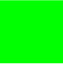) or 50 μg/kg (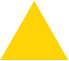) Fgl1-treated CIA mouse model. The values are mean ± SEM, and the statistical significance was calculated by One-way ANOVA. The asterisks indicate a significant difference. NS, not significant; *P < 0.05; **P < 0.01; ***P < 0.001. Black arrow, articular cartilage. Representative histopathology images at 20× magnification. Bars = 200 μm.

**Figure 5:**
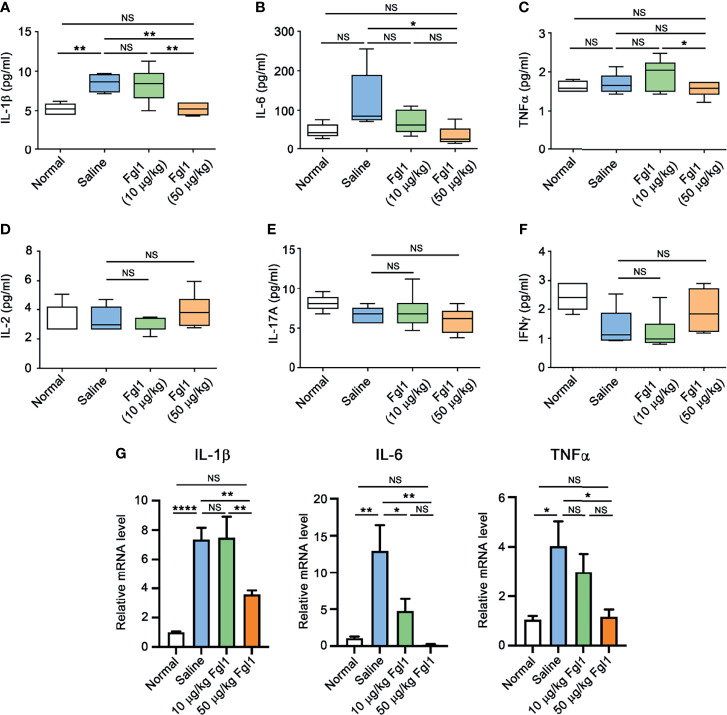
Expression level of pro-inflammatory cytokines in paw tissue of Fgl1-treated CIA mice. Hind paw samples were collected from normal DBA/1J mice (Normal, n=5) or CIA mice treated with saline (n=5), 10 (n=6) or 50 μg/kg (n=6) Fgl1 protein at day 42 following the complete therapeutic regimen. Paw tissue were homogenized in lysis buffer containing protease inhibitors. The protein levels of **(A)** IL-1β, **(B)** IL-6, **(C)** TNFα, **(D)** IL-2, **(E)** IL-17A and **(F)** IFNγ were detected and quantified by MILLIPLEX MAP magnetic bead panel assay. **(G)** The mRNA levels of IL-1β, IL-6 and TNFα were analyzed by real time qPCR. All data are the mean ± SEM of at least pentaplicate independent experiments. Statistical significance was calculated by One-way ANOVA. NS, not significant; *P < 0.05; **P < 0.01; ****P < 0.0001 when compared between each indicated group.

In order to evaluate the therapeutic potential of Fgl1 for RA, we established a collagen-induced arthritis (CIA) model using DBA/1J mice (**Figure 3A**) and administrated normal saline or different concentrations of Fgl1 recombinant protein (10 or 50 μg/kg) at day 24, at which time point arthritis had already begun (arthritis score exceeded 1). As shown in **Figure 3B**, arthritis was suppressed in a dose-dependent manner upon treatment with Fgl1 from day 24. Arthritis on day 42 was inhibited 22% and 62.5%, by 10 and 50 μg/kg of Fgl1, respectively, as compared with the normal saline-treated control group (**Figure 3B**). **Figure 3C** further shows that the hind paw of normal saline-treated mice or low-dosage Fgl1-treated group (i.e., 10 μg/kg) was obviously swollen and red as compared with normal DBA/1J mice, and the inflammatory effect was significantly reduced in the high-dosage Fgl1-treated group (i.e., 50 μg/kg) (**Figure 3C**). Histological analysis was performed on the hind leg of each mouse at the end of the experiment. As shown in **Figures 3D–G**, the normal saline-treated CIA mouse model revealed severe pathological signs of arthritis such as inflammation, cellular infiltration or bone deformation as compared with normal DBA/1J mice (**Figures 3D, E**). And mice receiving high-dosage of Fgl1 had significantly less infiltration of inflammatory cells and bone deformation in the joint tissue than normal saline-treated CIA mice (**Figures 3F, G**). Cartilage destruction was analyzed in the joint tissue and quantified using sections stained with toluidine blue (**Figure 4**). Results show that the cartilage damage was significant lesser in high-dosage of Fgl1 treated group as compared with saline-treated control group. The average histopathological score, bone erosion score and cartilage damage score in the groups are shown in **Figures 3H, I** and **4B**, respectively. Moreover, we analyzed the protein and mRNA level of local cytokines involved in inflammation, such as IFNγ, IL-1β, IL-2, IL-6, IL-17A and TNFα, in the hind leg by multiplex cytokine assay and real-time qPCR, respectively. **Figure 5** shows that both pivotal pro-inflammatory cytokines, IL-1β and IL-6, were significantly decreased after treatment with high-dosage of Fgl1. Cytokine IL-1β decreased from 8.520 ± 0.4912 pg/ml in the normal saline-treated CIA mice to 5.190 ± 0.3075 pg/ml in the high-dosage-treated group (*P* < 0.01) (**Figure 5A**). Cytokine IL-6 also decreased from 122.4 ± 34.26 pg/ml in the normal saline-treated CIA mice to 34.70 ± 9.347 pg/ml in high-dosage-treated group (*P* < 0.05) (**Figure 5B**). In contrast, there was no significant difference on the concentration of TNFα, IL-2, IL-17A and IFNγ between normal saline- and Fgl1-treated groups (**Figures 5C–F**). Real-time qPCR data also showed that the mRNA expression levels of IL-1β, IL-6 and TNFα were significant decreased in high-dosage Fgl1-treated group as compared with saline-treated control group (**Figure 5G**). However, the expression level of IL-2, IL-17A and IFNγ was too low to be determined (data not shown). These results suggested that Fgl1 can attenuate collagen-induced arthritis progression, decrease pathological signs and part of pro-inflammatory cytokine levels in the joint region of the CIA mouse model.

### Potential Adverse Events in the Fgl1-Treated CIA Mouse Model

**Figure 6:**
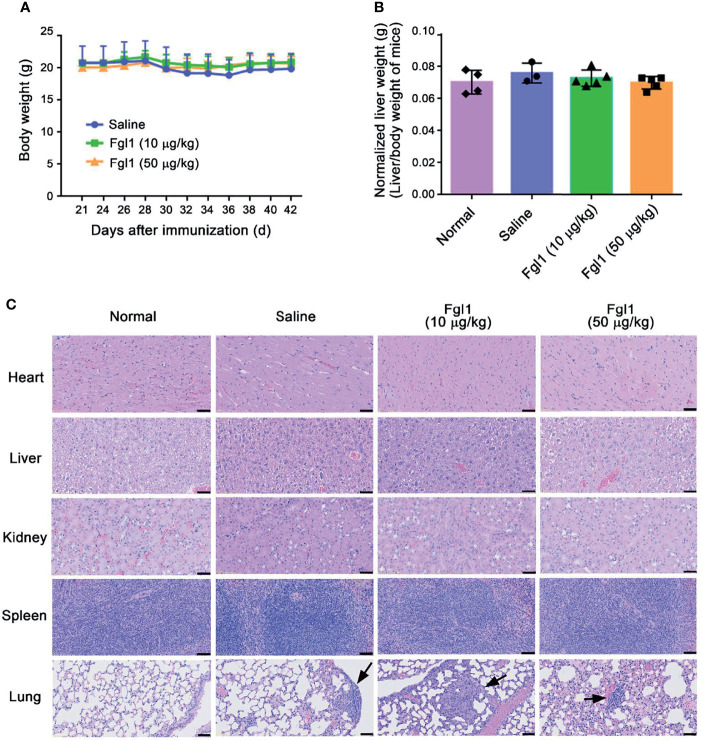
Potential adverse events of Fgl1 treatment in CIA mice. **(A)** Body weights of the mice in each group were monitored every 2 days after treatment with normal saline (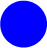), 10 (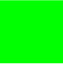) or 50 μg/kg (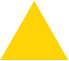) Fgl1 protein. Multiple organ samples (i.e., heart, liver, kidney, spleen and lung) were collected from normal DBA/1J mice or CIA mice treated with saline, or 10 or 50 μg/kg Fgl1 protein at day 42 following the complete therapeutic regimen. **(B)** Liver weight of mice from each group was measured and normalized to their own body weight. **(C)** Representative HE stained tissue sections of the heart, liver, kidney, spleen and lung in normal DBA/1J mice, CIA mouse model after treatment with normal saline, or 10 or 50 μg/kg Fgl1 protein. Black arrow, cellular infiltration. Representative histopathology images at 10× magnification. Bars = 50 μm.

In order to examine whether Fgl1 treatment will cause potential adverse events based on its physiological function [i.e., inducing hepatocyte proliferation (12–14)], we monitored the body weight of each mouse over the duration of therapy and further weighed the livers at the end of the experiment. As shown in **Figures 6A, B**, there was no significant difference in body weight (**Figure 6A**) and normalized liver weight (**Figure 6B**) in the Fgl1- and saline-treated groups. Histological staining of several organs including heart, liver, spleen, lung and kidney was further performed to analyze the diversification of tissue morphology. As shown in **Figure 6C**, there was no significant change in histological morphology of most of organs, including heart, liver, spleen and kidney, observed after treating with Fgl1 recombinant protein as compared with normal control mice. In contrast, the cellular infiltration of lung was observed in normal saline- and Fgl1-treated CIA mouse model as compared with normal DBA/1J mice (**Figure 6C**). It may cause by the anti‐citrullinated protein antibodies (ACPAs) can interact with various citrullinated proteins that not only be generated at joints, but also in the lung, thereby inducing RA‐associated lung inflammation (20, 21). Collectively, these results indicate that Fgl1 treatment may not cause obvious toxicity to normal organs.

## Discussion

In this study, we evaluated the therapeutic effect of Fgl1 on arthritis progression in a collagen-induced arthritis (CIA) mouse model. We first confirmed that Fgl1 can specifically bind to the surface Lag-3 receptor, which is a newly-identified immune checkpoint receptor for Fgl1 (15), and further inhibit T cell activation. Intraperitoneal administration of Fgl1 protein can significantly decrease inflammatory cytokine level in local paw tissue, prevent joint inflammation, cellular infiltration and bone deformation and attenuate collagen-induced arthritis progression *in vivo*. We also proved that exogenous Fgl1 will not cause obvious adverse events during treatment by monitoring body weight, liver weight and assessing phenotype of several organs (e.g., heart, liver, spleen, kidney and lung) by pathological study. We thus expect that Fgl1 protein may serve as a potential therapeutic agent for RA therapy in the future.

Lymphocyte-activation gene 3 (LAG-3) is an important inhibitory immune checkpoint protein that is expressed on the surface of activated CD4^+^ T cells, CD8^+^ T cells, natural killer T cells (NKT) and NK cells (22–24) and also plays a critical role in regulation of immune system homeostasis (24, 25). Loss-of-function of LAG-3 may cause unlimited T cell activation, proliferation and accelerate the progression of autoimmune diseases. Bettini and colleagues indicated that absence of LAG-3 increased islet antigen (Ag)-specific T cell infiltrate into pancreatic islets of non-obese diabetic (NOD) mice at a younger age and they developed significantly accelerated diabetes with 100% incidence (26). Angin et al. further suggested that treatment with LAG-3-specific humanized agonist Ab, IMP761, can inhibit T cell receptor (TCR)-mediated nuclear factor of activated T-cell (NFAT) activation, and Ag-induced human T cell proliferation and activation, thereby suppressing the symptoms (e.g., cellular infiltration or erythema) of delayed-type hypersensitivity (DTH) in the cynomolgus macaque (27), indicating that LAG-3 signaling is important to suppress autoimmune disorders. In our study, we also proved that Fgl1, the new-found major ligand of LAG-3 (15), can significantly decrease inflammatory cytokine level in local paw tissue (**Figure 5**), prevent joint inflammation, cellular infiltration and bone deformation and attenuate arthritis progression in a CIA mouse model (**Figure 3D**). Thus, we expect that an effective inhibitory ligand (e.g., Fgl1) for LAG-3 receptor or LAG-3 specific agonist (e.g., IMP761) may be potential therapeutic agent for different types of T cell-induced autoimmune or inflammatory diseases.

Several binding ligands of LAG-3, including major histocompatibility complex (MHC) class II (MHC-II) (28), galectin-3 (Gal-3) (29, 30), LSECtin (31) and FGL1 (15) have been identified over the past three decades. They all bind to LAG-3 with independent epitopes and suppress T cell activation through different mechanisms. The canonical ligand for LAG-3 is MHC-II, once MHC-II interacts with the extra loop of the D1 domain of LAG-3, an unclear inhibitory signal is delivered to effector T cells through cytoplasmic domain of LAG-3, thereby suppressing T cell activity (28). Gal-3 and LSECtin transmit a negative signal by binding to the glycosylated site on LAG-3 (29–31). In contrast, Wang et al. suggested that FGL1 can suppress T cell activity after interacting with D1-D2 domain of LAG-3 by its fibrinogen like domain (15) and the MHC-II-independent binding manner implies that FGL1 may perform co-inhibition of activated T cells by cooperating with other inhibitory ligands of LAG-3 without mutual interference. We thus expect that FGL1 will be suitable to serve as a combinatory therapeutic agent with other potential inhibitory ligand drugs in autoimmune diseases.

Disruption of the biological function of pro-inflammatory cytokines is the major therapeutic strategy for RA therapy. Several pro-inflammatory cytokines (e.g., TNFα, IL-1β and IL-6) were observed in synovial fluid of RA patients and correlated with the severity of RA disease (32). Cytokines, especially TNFα, produced by infiltrated inflammatory cells in joints are involved in RA progression. Brennan et al. demonstrated that treatment with TNFα neutralizing antibody can significantly suppress the expression of other pro-inflammatory cytokines in inflamed RA joints, such as IL-1β, IL-6, IL-8 and granulocyte monocyte-colony stimulating factor (GM-CSF), and attenuate pathological signs in the RA mouse model (33–37). Our results also showed that both of IL-1β and IL-6 in inflamed paw tissue, were significantly decreased from 8.520 ± 0.4912 to 5.190 ± 0.3075 pg/ml and 122.4 ± 34.26 to 34.70 ± 9.347 pg/ml, respectively, after treatment with a high-dosage of Fgl1 (i.e. 50 μg/kg) (**Figures 5A, B**). However, there was no significant difference observed in TNFα level between the groups. This may be attributed to the time point of tissue sampling in our experimental procedure because TNFα participates in the initial inflammatory process of RA disease (38, 39). Collectively, Fgl1 treatment may potentially inhibit critical pro-inflammatory biomarkers of RA through suppression of T cell activity, thereby attenuating the pathological signs of RA disease in the CIA mouse model.

Long-termly and systemic administration of FGL1 to arthritis model may increase the risk of unpredictable effect during treatment. FGL1 serves as an acute phase protein that secreted by liver upon liver injury (12, 13) and plays important role in several physiological functions such as promotion of hepatocyte proliferation, regulation of lipid metabolism and energy utilization (40). Although there was no obvious toxicity being observed in this study (**Figure 6**), however, collagen-induced arthritis (CIA) mouse model, which was used in this study, do not mimic the chronic nature of RA disease in patients and hard to be used to evaluate the safety of long-term systemic FGL1 treatment. In the future, a chronic arthritis mouse model, which is established by injecting cocktail of cartilage-specific antibodies and appropriate adjuvant (e.g. lipopolysaccharide or mannan) (41), will be used to investigate whether any potential adverse effects happened and further evaluate the therapeutic safety of long-term systemic FGL1 treatment.

## Conclusion

In summary, this study evaluated the therapeutic efficacy of FGL1 protein on the autoimmune disease RA. We demonstrated that Fgl1 can specifically bind to Lag-3 receptor and inhibit T cell activation *in vitro*. Treatment with Fgl1 further attenuated local inflammation, cellular infiltration, bone deformation and clinical score of arthritis in a CIA mouse model with minimal adverse events. We believe that Fgl1 protein may serve as a potential therapeutic agent for RA or other T cell-mediated autoimmune disease therapy in the future.

## Data Availability Statement

The original contributions presented in the study are included in the article/**Supplementary Material**. Further inquiries can be directed to the corresponding authors.

## Ethics Statement

Animal experiments were carried out in accordance with institutional guidelines and reviewed and approved by the Animal Care and Use Committee of Kaohsiung Medical University, Kaohsiung, Taiwan (IACUC: 106117).

## Author Contributions

W-WL, K-WH, H-HS, and T-FF performed the experiments. W-WL, T-FF, M-SC, Y-CT, E-SL, and H-KH interpreted the results. W-WL, T-FF, and H-KH wrote the manuscript. W-WL, K-WH, and T-LC designed the experiments and draw the figures. I-JC, Y-CL, Y–CS, Y-TW, and S-CT assisted in manuscript editing and proofreading. All authors read and approved the final manuscript.

## Funding

This work was supported by grants from the Ministry of Science and Technology, Taiwan (MOST-110-2628-B-037-010); the KMU-KMUH Co-Project of Key Research (KMU-DK(B)110006) and Research Foundation (KMU-KI110004) from Kaohsiung Medical University, Taiwan; NSYSU-KMU joint research project (NSYSUKMU 110-I002); the Medical Research Fund of Kaohsiung Armed Forces General Hospital (802KB109388).

## Conflict of Interest

The authors declare that the research was conducted in the absence of any commercial or financial relationships that could be construed as a potential conflict of interest.

## Publisher’s Note

All claims expressed in this article are solely those of the authors and do not necessarily represent those of their affiliated organizations, or those of the publisher, the editors and the reviewers. Any product that may be evaluated in this article, or claim that may be made by its manufacturer, is not guaranteed or endorsed by the publisher.

## Supplementary Material

Supplementary Figure 1 SDS-PAGE and Western blot for commercial histidine-tagged mouse Fgl1 protein. **(A)** SDS-PAGE analysis of histidine-tagged mouse Fgl1 protein (mFgl1-his): lane 1, molecular weight marker; lane 2, mFgl1-his. The mFgl1-his protein was detected with **(B)** anti-his Ab or **(C)** anti-mouse Fgl1 Ab and HRP-conjugated secondary Ab by Western blot. Lane 1, molecular weight marker; lane 2, mFgl1-his. The black arrow indicates the mFgl1-his protein (approximately 35.5 kDa).

Supplementary Figure 2 Fgl1 treatment has no effect on T cell proliferation. Primary mouse T cells were labeled with carboxyfluorescein diacetate succinimidyl ester (CFSE), then incubated with anti-CD3/anti-CD28 antibody-coated beads and cultured in the presence or absence of Fgl1 recombinant protein (5 μg/ml) for 3 days. The fluorescent signal was detected by flow cytometry. Gray, cell alone; Red, CFSE-labeled primary mouse T cells were stimulated with anti-CD3/anti-CD28 antibody-coated beads; Blue, CFSE-labeled primary mouse T cells was co-incubated with anti-CD3/anti-CD28 antibody-coated beads and Fgl1 protein.

Supplementary Table 1 List of oligonucleotides for real-time PCR.
