# Supplementary material for: Fibrinogen-Like Protein 1 Serves as an Anti-Inflammatory Agent for Collagen-Induced Arthritis Therapy in Mice
Source: Front Immunol. 2021 Dec 16;12:767868. doi: 10.3389/fimmu.2021.767868 (PMC8716738; doi:10.3389/fimmu.2021.767868)

Supplementary information

Materials and Methods

Western blot and SDS-PAGE

One microgram of histidine-tagged mouse Fgl1 protein (mFgl1-his) was mixed with 6 × SDS reducing loading dye and boiled for 10 min. Samples were separated by 10% SDS-PAGE and then transferred to nitrocellulose (NC) membranes (Millipore, Billerica, MA, USA). After blocking with phosphate-buffered saline (PBS) containing 5% milk at 4 °C overnight, the membranes were incubated with mouse anti-his Ab (Millipore, Billerica, MA, USA) or rabbit anti-mouse Fgl1 Ab (OriGene Technologies, Rockville, MD, USA), respectively, at room temperature (RT) for 1 h. After washing, the membrane were incubated with HRP-conjugated goat anti-mouse IgG Fc or HRP-conjugated goat anti-rabbit IgG Fc antibodies (Jackson ImmunoResearch Laboratories, West Grove, PA, USA), respectively, at RT for 1 h. After extensive washing, the blots were visualized by enhanced chemiluminescence detection according to the manufacturer's instructions (Merck Millipore). To analyze the molecular weight (MW) and purity of purified mFgl1-his, 2 μg of recombinant protein was separated by 10% SDS-PAGE and then stained with Coomassie Brilliant Blue.

Proliferation assay of Fgl1-treated T cells

Mouse T cells were isolated from splenocytes of wild-type female BALB/c mice using Pan T cell isolation kit, a LS Column and a MidiMACS Separator (Miltenyi Biotec, Bergisch Gladbach, North Rhine-Westphalia, Germany). Isolated T cells (1×10^6) were labelled with CFSE (2 μM) for 10 min, washed, then activated by incubating with anti-mouse CD3 and anti-mouse CD28 antibody-coated beads in the presence or absence of 5 μg/ml Fgl1 recombinant protein and cultured in a 48-well plate at 37°C for 3 days. Cell proliferation was analyzed by flow cytometry after centrifugation, and the generations were analyzed by guava easyCyte flow cytometry system (Merck, Kenilworth, NJ, USA).

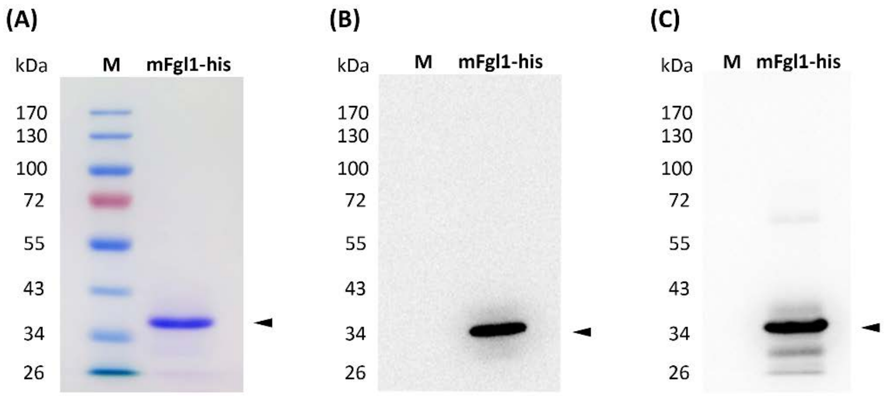

Supplementary Figure 1. SDS-PAGE and Western blot for commercial histidine-tagged mouse Fgl1 protein. (A) SDS-PAGE analysis of histidine-tagged mouse Fgl1 protein (mFgl1-his): lane 1, molecular weight marker; lane 2, mFgl1-his. The mFgl1-his protein was detected with (B) anti-his Ab or (C) anti-mouse Fgl1 Ab and HRP-conjugated secondary Ab by Western blot. Lane 1, molecular weight marker; lane 2, mFgl1-his. The black arrow indicates the mFgl1-his protein (approximately 35.5 kDa).

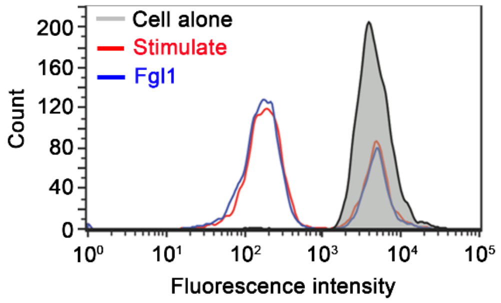

Supplementary Figure 2. Fgl1 treatment has no effect on T cell proliferation. Primary mouse T cells were labeled with carboxyfluorescein diacetate succinimidyl ester (CFSE), then incubated with anti-CD3/anti-CD28 antibody-coated beads and cultured in the presence or absence of Fgl1 recombinant protein (5 µg/ml) for 3 days. The fluorescent signal was detected by flow cytometry. Gray, cell alone; Red, CFSE-labeled primary mouse T cells were stimulated with anti-CD3/anti-CD28 antibody-coated beads; Blue, CFSE-labeled primary mouse T cells was co-incubated with anti-CD3/anti-CD28 antibody-coated beads and Fgl1 protein.

Supplementary Table 1: List of oligonucleotides for real-time PCR

**Gene**	**Sequence (5’ to 3’)**
IL-1β	F: AAGATGAAGGGCTGCTTCCA<br>R: GTGCTGCTGCGAGATTTGAA
IL-2	F: CCCAAGCAGGCCACAGAA<br>R: CGCAGAGGTCCAAGTTCATCT
IL-6	F: TCGGAGGCTTAATTACACATGTTC<br>R: TGCCATTGCACAACTCTTTTCT
TNFα	F: CCACCACGCTCTTCTGTCTACTG<br>R: CTGATGAGAGGGAGGCCATTT
IL-17A	F: CAGGACGCGCAAACATGA<br>R: GCAACAGCATCAGAGACACAGAT
IFNγ	F: CCACGGCACAGTCATTGAAA<br>R: TTCCACATCTATGCCACTTGAGTT

Original pictures of Supplementary Figure 1

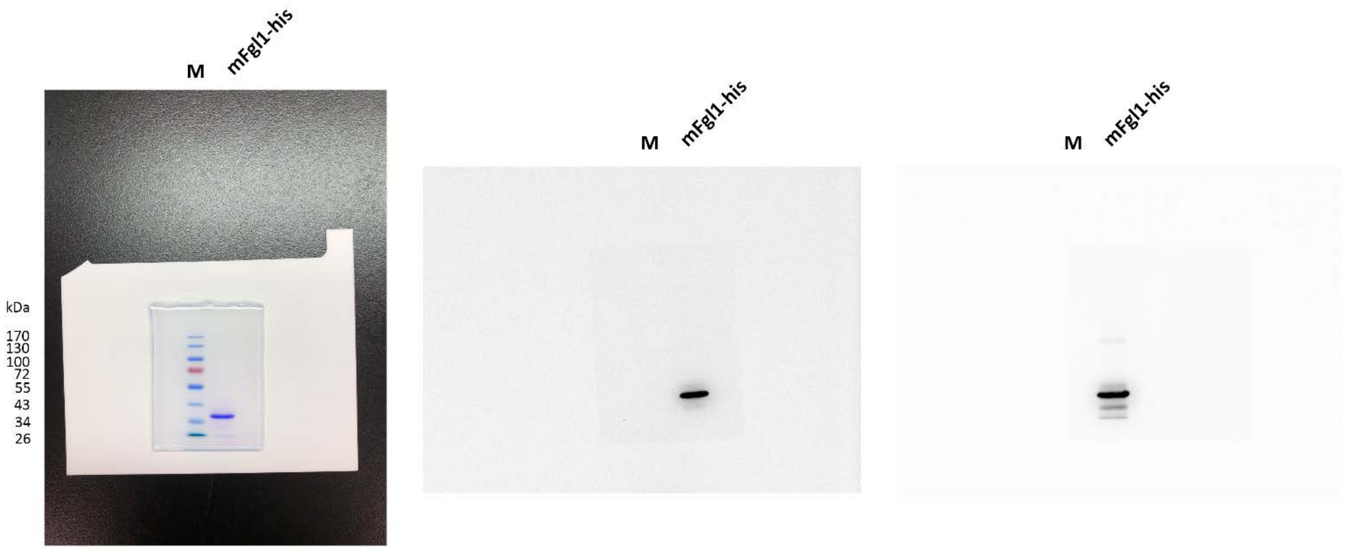

Supplement: Supplementary Figure 1 — SDS-PAGE and Western blot for commercial histidine-tagged mouse Fgl1 protein. (A) SDS-PAGE analysis of histidine-tagged mouse Fgl1 protein (mFgl1-his): lane 1, molecular weight marker; lane 2, mFgl1-his. The mFgl1-his protein was detected with (B) anti-his Ab or (C) anti-mouse Fgl1 Ab and HRP-conjugated secondary Ab by Western blot. Lane 1, molecular weight marker; lane 2, mFgl1-his. The black arrow indicates the mFgl1-his protein (approximately 35.5 kDa). [file DataSheet_1.pdf]
